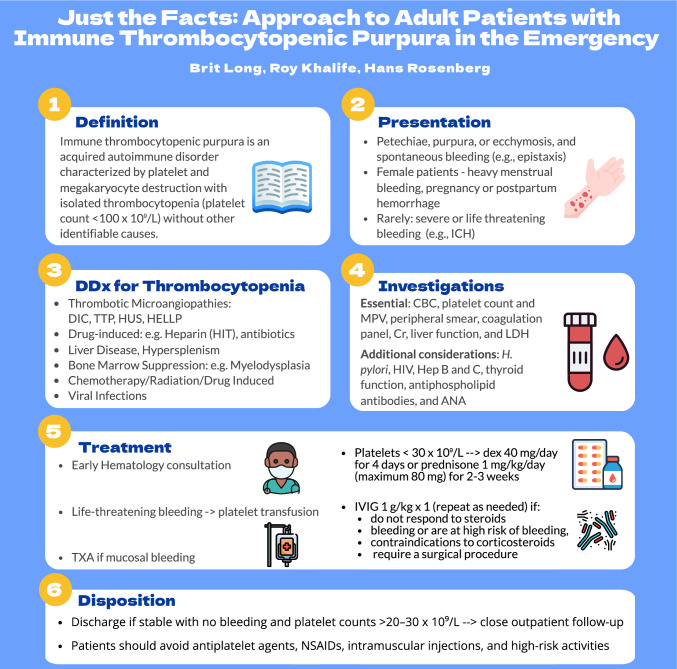# Just the facts: approach to adult patients with immune thrombocytopenic purpura in the emergency department

**DOI:** 10.1007/s43678-025-01024-y

**Published:** 2025-10-23

**Authors:** Brit Long, Roy Khalife, Hans Rosenberg

**Affiliations:** 1 Department of Emergency Medicine, University of Virginia Medical Center, Charlottesville, VA USA; 2 Division of Hematology, Department of Medicine, University of Ottawa, Ottawa, ON Canada; 3 Department of Emergency Medicine, University of Ottawa, Ottawa, ON Canada

**Keywords:** Hematology, Immune thrombocytopenic purpura (ITP), Bleeding, Purpura, Thrombocytopenia, Corticosteroids, Intravenous immunoglobulin (IVIG), Hématologie, purpura thrombopénique immunologique (PTI), Saignement, Purpura, Thrombocytopénie, Corticostéroïdes, Immunoglobuline intraveineuse (IgIV)

## Clinical scenario

*A 32-year-old woman presents to the emergency department (ED) with a 2-day history of petechial rash on her legs and easy bruising. She denies trauma, recent illness, or medication use. Her vital signs are stable. On examination, she has no active bleeding, but scattered petechiae are noted on her lower limbs with wet purpura along the mucosal surface of the right cheek.*

## What is immune thrombocytopenic purpura, and who is typically affected?

Immune thrombocytopenic purpura (ITP) is an acquired autoimmune disorder characterized by platelet and megakaryocyte destruction with isolated thrombocytopenia (platelet count < 100 × 10⁹/L) without other identifiable causes [1–4]. ITP may be primary (idiopathic) or secondary, associated with other diseases, such as infection (e.g., human immunodeficiency virus, hepatitis B or C, and *Helicobacter pylori*), autoimmune conditions (e.g., systemic lupus erythematosus, rheumatoid arthritis, and antiphospholipid syndrome), lymphoproliferative disorders (e.g., chronic lymphocytic anemia), immunodeficiency states (e.g., common variable immunodeficiency and selective IgA deficiency) or medications (e.g., quinine, acetaminophen, abciximab, carbamazepine, rifampicin, and vancomycin) [2, 3]. The incidence ranges between 2 and 5 cases per 100,000 person-years, with a prevalence ranging between 9 and 24 per 100,000 [2, 3]. There are three peaks: first in children between 2 and 10 years (peak incidence at 1–5 years), a second peak between 20 and 30 years with a female predominance, and the third peak in those older than 60 years with equal distribution between men and women [1, 5–7]. In adults, ITP becomes a chronic disorder in approximately 75% of cases. In elderly patients, other causes for thrombocytopenia such as myelodysplastic syndrome or marrow infiltration should be considered, particularly if other lines are affected [2, 5].

## How do patients with immune thrombocytopenic purpura present?

The most common presenting symptoms include petechiae, purpura, or ecchymosis, as well as spontaneous bleeding (e.g., epistaxis or gingival bleeding), particularly in those with platelet counts < 20–30 × 10⁹/L [1–4]. Female patients may experience heavy menstrual bleeding or postpartum hemorrhage. Severe bleeding (e.g., intracranial hemorrhage) may occur but is rare in patients with platelet counts > 10 × 10⁹/L. Generalized fatigue can occur in up to half patients with ITP [2, 3]. Fever, night sweats, weight loss, abdominal distension/tenderness, hepatosplenomegaly, lymphadenopathy, focal neurologic deficits, altered mental status, cardiac symptoms, arthralgias, and mouth ulcers should not be present in ITP, and these raise concern for another condition [2, 3].

## What investigations are needed in the ED for suspected immune thrombocytopenic purpura?

There is no diagnostic laboratory test for ITP, and the diagnosis is based on a platelet count < 100 × 10⁹/L while excluding other conditions. Essential laboratory testing in the ED setting includes complete blood cell count (platelet count to confirm isolated thrombocytopenia and mean platelet volume which if elevated could suggest a congenital cause for the thrombocytopenia), peripheral smear (exclude platelet clumping or pseudothrombocytopenia, schistocytes, malignant blood cells, parasitic infections, etc.), coagulation panel (exclude disseminated intravascular coagulation, advanced liver disease, and other bleeding diathesis), renal/liver function (exclude secondary causes and assess for comorbidities), and lactate dehydrogenase (exclude thrombotic microangiopathies, high-grade lymphoproliferative neoplasms, or other hematology malignancies) [2, 3]. A pregnancy test should be obtained in females as ITP may occur in 2–3% of pregnancies at any trimester. Additional non-urgent testing to assess for secondary causes may include evaluation for human immunodeficiency virus, *H. pylori*, hepatitis B and C, thyroid function, antiphospholipid antibodies, and antinuclear antibody tests. Bone-marrow examination may be considered in those who do not respond to ITP-directed therapies or if there are features suggestive of alternate etiology such as hematology malignancies [2, 3]. Patients with neurologic changes require emergent head computed tomography to assess for intracranial hemorrhage.

## What are the emergency management considerations in immune thrombocytopenic purpura?

The emergency clinician should assess for mucosal bleeding; menorrhagia, and critical bleeding. This is defined as bleeding in an anatomical site, including intracranial, intraspinal, intraocular, retroperitoneal, pericardial, or intramuscular with compartment syndrome; or ongoing bleeding resulting in hemodynamic instability or respiratory compromise [8]. This definition is intended for patients with suspected or confirmed severe ITP (platelet count < 20 × 10⁹/L) [8]. Hematology consultation is recommended to assist with further testing and treatment decisions. The primary goals of management include stopping active bleeding and reducing the risk of future bleeding [2–4, 8]. In patients with platelet count < 20–30 × 10⁹/L and active bleeding, treatment with corticosteroids plus intravenous immunoglobulin is recommended, along with admission for observation and to ensure that the platelet count rises > 30 × 10⁹/L [2, 3, 8, 9]. In those with life-threatening bleeding (e.g., intracranial hemorrhage), emergent platelet transfusion is recommended. Antifibrinolytic treatment with tranexamic acid may be utilized in patients with significant mucosal bleeding. For those with platelet count < 30 × 10⁹/L and no active bleeding, corticosteroids should be initiated, and the patient may be discharged with hematology follow-up if the patient is otherwise stable. If the platelet count is > 30 × 10⁹/L, the patient should follow-up with hematology, but corticosteroids are not necessary [2, 3, 9].

Corticosteroids typically include dexamethasone (40 mg/day for 4 days) or prednisone 1 mg/kg/day (maximum 80 mg) for 2–3 weeks followed by taper [2, 3, 9]. Of note, a 2016 meta-analysis found that dexamethasone is associated with faster response at 14 days, greater platelet improvement, and less toxicity [10]. Up to 80% of patients respond to corticosteroids, typically within 2–3 weeks, and 30–50% of adults will demonstrate a sustained response [2, 3, 9]. Intravenous immunoglobulin may be used in those who are bleeding or are at high risk of bleeding, do not respond to corticosteroids, have contraindications to corticosteroid therapy (e.g., insulin-dependent or uncontrolled diabetes and psychiatric disorders), or if requiring a surgical procedure. One dose of intravenous immunoglobulin 1 g/kg is effective in up to 80% of cases, with a response typically within 24–48 h, peak platelet level within 72 h, and an effect lasting approximately 2 weeks [2, 3, 9]. If corticosteroids and intravenous immunoglobulin are ineffective, further treatments include thrombopoietin receptor antagonists, rituximab, fostamatinib, and splenectomy, and further hematology specialist testing may be required [2–4, 9].

## What are the key disposition and follow-up considerations?

Discharge is reasonable in stable patients with no bleeding, with close outpatient hematology follow-up arranged [2–4]. Patients not meeting these criteria should be admitted to a center with access to a hematology specialist.

Patients should avoid antiplatelet agents, quinine, non-steroid anti-inflammatory drugs (except celecoxib), and other medications associated with thrombocytopenia, as well as intramuscular injections and high-risk activities (e.g., high-impact sports, motorcycle riding, and rock climbing). Anticoagulation should be avoided unless the benefits outweigh the bleeding risk, particularly in the context of significant thrombocytopenia or bleeding.

## Case resolution

*Her platelet count returns at 8* × *10⁹/L. Smear confirms isolated thrombocytopenia with large platelets, but there is no evidence of hemolysis. She receives dexamethasone and intravenous immunoglobulin in the ED, and hematology is consulted. She remains hemodynamically stable and is admitted.*